# The Epidemiological Characteristics of Noncommunicable Diseases and Malignant Tumors in Guiyang, China: Cross-sectional Study

**DOI:** 10.2196/36523

**Published:** 2022-10-28

**Authors:** Qibing Zeng, Jingyuan Yang, Ziyun Wang, Haiyan Liu, Junhua Wang, Tingting Yang, Jin Hu, Han Guan, Yun Lu, Huijuan Liu, Feng Hong

**Affiliations:** 1 The Key Laboratory of Environmental Pollution Monitoring and Disease Control Ministry of Education & School of Public Health Guizhou Medical University Guiyang China

**Keywords:** epidemiological characteristics, noncommunicable diseases, malignant tumors, cross-sectional study, Guiyang

## Abstract

**Background:**

Studies that address the changing characteristics of diseases are of great importance for preventing and controlling the occurrence and development of diseases and for improving health. However, studies of the epidemiological characteristics of noncommunicable diseases (NCDs) and malignant tumors (MTs) of the residents in Guiyang, China, are lacking.

**Objective:**

The aim of this study was to evaluate the prevalences of NCDs and MTs in residents of Guiyang, Guizhou Province, China, and analyze differences among ages, genders, and regions.

**Methods:**

A multistage stratified cluster sampling method was used. Based on the inclusion and exclusion criteria, 81,517 individuals were selected for the study. Of these, 77,381 (94.9%) participants completed the study. Structured questionnaires were used to collect information on demographic characteristics, NCDs, and MTs. The chi-square test (with 95% confidence intervals) was used to analyze differences in disease prevalence among genders, ages, and geographical regions.

**Results:**

The major chronic NCDs of Guiyang residents are obesity, hypertension, and diabetes. MTs in women are mostly breast cancer, cervical cancer, and endometrial cancer, whereas in men, MTs are mainly lung cancer, rectal cancer, and gastric cancer. The prevalences of hypertension and diabetes in women are higher than in men, but the prevalences of lung cancer and gastric cancer in men are higher than in women. The epidemiological characteristics of individuals in different life stages are dissimilar. In terms of regional distribution, the prevalences of the above diseases in the Baiyun and Yunyan districts of Guiyang are relatively high.

**Conclusions:**

Several NCDs (obesity, hypertension, and diabetes) and MTs (women: breast cancer, cervical cancer, and endometrial cancer; men: lung cancer, rectal cancer, and gastric cancer) should be the focus for the prevention and control of chronic diseases in the future. In particular, the Baiyun and Yunyan districts of Guiyang are the important regions to emphasize.

## Introduction

Noncommunicable diseases (NCDs) are a general term for a class of diseases with insidious onset, long course, and complex etiology—some of which have not been fully confirmed. NCDs are some of the main health challenges of the 21st century, posing a great threat to human survival and economic development [1,2]. Epidemiological studies have shown that NCDs are on the rise in low-income countries and have led to higher mortality rates in these countries [3,4]. According to incomplete statistics, NCDs now account for more than one-half of the global burden of disease [5]. In 2020, NCDs were estimated to account for 80% of the global disease burden and 7 out of 10 deaths in low- and middle-income countries [6]. Therefore, the prevention and control of the pandemic of chronic NCDs has become a major scientific issue that needs urgent solutions to allow sustainable development while reducing disease burden in countries around the world [7-10].

Disease prevention is the most important means for promoting the health of the population, and although its purpose is to eliminate diseases, if diseases cannot be eliminated, disease prevention can still minimize the harm of diseases to the human body, thereby ensuring and improving the health of the population, social progress, and economic sustainability and continuous development [11]. However, in determining how to effectively implement disease prevention and control measures, the most critical issue is to grasp the epidemiology and characteristics of diseases in the population, among which disease spectrum investigation is one of the most important ways for exploring the prevalence of diseases [12].

Guiyang is one of the most distinctive regions in Southwest China, with highly heterogeneous genetic variation, cultural background, socioeconomic status, and geographical environment. With the continuous development of the economy and the increasing enrichment of lifestyle, the problem of resident disease burden has received continuous attention. However, studies of the epidemiological characteristics of NCDs and malignant tumors (MTs) of the residents in Guiyang, China, are lacking. The aim of this study was to evaluate the prevalences of NCDs and MTs in Guiyang, China, and analyze differences among ages, genders, and regions. To our knowledge, this is the first study of its kind. The study findings will aid the government in developing cost-effective and targeted local resource allocation and other preventive health policies that address NCDs, MTs, and related health inequities.

## Methods

### Study Participants

We conducted a cross-sectional study in Guiyang, China, from January 2017 to December 2017. We worked with the local centers for disease control and prevention, clinical centers, and local government to recruit volunteers. According to economic, geographical, population, and other factors in combination with the sample size requirement of the study design, a total of 81,517 individuals were selected as study participants by multistage stratified cluster sampling. Based on data from China’s sixth census, the number of people in this study accounted for 3.52% of the population of the sampling area and 1.88% of the total population of Guiyang. The specific sampling method, the distribution of study participants, and the on-site investigation procedure are detailed in Multimedia Appendices 1-3, respectively.

Study selection criteria were as follows: (1) informed consent was received from the participant; and (2) the participant belonged to the permanent population of Guiyang, China (defined as either a member of the migrant population who lived in the city for 6 of the past 12 months or a member of the local population who has been away from the city for less than 12 months). Individuals in different stages of life were eligible, as there were no specific age requirements for study participation. Study exclusion criteria were (1) inability to provide a unique national identity card, (2) presence of a severe physical or mental illness (such as schizophrenia or bipolar disorder), and (3) refusal to comply with research requirements.

### Ethics Approval

The content, protocol, informed consent, and recruitment materials of this study were approved by the ethics committee of the affiliated hospital of Guizhou Medical University (NO.2017ER67). Written informed consent was obtained from all participants before data collection.

### Questionnaire Interview

A structured questionnaire was used to record information about demographic factors, health behavior, and the disease history of the participants (see more details in Multimedia Appendix 4). The information was collected during a face-to-face interview, conducted by well-trained interviewers recruited from students with a medical background at Guizhou Medical University. The specific procedure and methods of the questionnaire interview are detailed in Multimedia Appendices 1 and 4, respectively.

We also instituted a series of quality control measures including designing logical constraints into the questionnaire to avoid errors in logic, noting the time spent on each questionnaire, and randomly sampling questionnaire responses on the interview day to evaluate the data quality (ie, the sampling plan ensured that each interviewer had at least been sampled once). Questionnaires that were classified as unqualified were excluded from the final analysis.

### Clinical Examination

We conducted medical examinations of the participants using mainly the resources and personnel at local clinical centers. To unify the data standards across different sites, we implemented a standardized training program for doctors and nurses before the study began.

Physicians at local clinical centers performed physical examinations (including measurements of height, weight, blood pressure, waist circumference, and hip circumference) and clinical examinations and determined diagnoses. The specific methods of the physical examinations are detailed in the Multimedia Appendix 1. Except for self-declaration by the participants, all disease information must have been recorded in the local health management system, or the participants could present a hospital diagnosis certificate to confirm the diagnosis.

According to the health industry standard of the People’s Republic of China “criteria of weight for adults” (WS/*t* 428-2013) [13], all participants were stratified into 1 of 4 groups: underweight (BMI<18.5), normal weight (18.5≤BMI<24.0), overweight (24.0≤BMI<28.0), or obese (BMI≥28.0).

### Statistical Analysis

EpiData software (version 3.0; EpiData Association) was used to input the data. Statistical analysis (including prevalence and chi-square test) was performed using SPSS statistical software (version 22.0; IBM Corp). Pearson chi-square test (T≥5), continuity-adjusted chi-square test (1≤T<5), and Fisher exact test (T<1) were used to analyze differences in the prevalences of chronic NCDs or MTs in different genders and various age groups. Choropleth maps were produced within a geographic information system to visually examine geographical variations in the prevalences of chronic NCDs and MTs across Guiyang. Data illustrated in the choropleth maps were median prevalence estimates with 95% confidence intervals calculated from unadjusted logistic regressions. A threshold of *P<*.05 was set to determine any significant differences between the averages.

## Results

### Characteristics of the Study Participants

At the end of the study, a total of 77,381 (94.9%) participants of the original 81,517 selectees had completed the standardized, full-course questionnaire interview and clinical examination. The characteristics of the study participants are presented in Table 1.

**Table 1:** Characteristics of the study participants (n=77,381).

Characteristic		Participant, n (%)
**Area (n=77,381)**		
	Yunyan district	29,479 (38.1)
	Baiyun district	11,262 (14.55)
	Wudang district	12,808 (16.55)
	Xiuwen county	10,243 (13.24)
	Qingzhen city	13,589 (17.56)
**Age (years; n=77,292)^a^**		
	0-19	18,263 (23.63)
	20-29	10,330 (13.36)
	30-39	11,139 (14.41)
	40-49	11,951 (15.46)
	50-59	10,000 (12.94)
	60-69	8569 (11.09)
	≥70	7045 (9.11)
**Gender (n=77,369)^a^**		
	Male	37,492 (48.46)
	Female	39,877 (51.54)
**Ethnicity (n=77,334)^a^**		
	Han	65,205 (84.32)
	Buyi	4108 (5.31)
	Miao	2678 (3.46)
	Dong	517 (0.67)
	Yi	873 (1.13)
	Chuanqing	1433 (1.85)
	Other	2520 (3.26)
**Education (n=77,338)^a^**		
	Illiterate or semiliterate	20,447 (26.44)
	Primary school	14,577 (18.85)
	Middle school	21,291 (27.53)
	High school	6618 (8.56)
	Technical secondary school or secondary vocational technical school	4270 (5.52)
	Junior college	5008 (6.48)
	Bachelor’s degree or above	5127 (6.63)
**Occupation (n=77,261)^a^**		
	Unemployed	13,075 (16.92)
	Farmer	16,095 (20.83)
	Civil servant	1080 (1.4)
	Medical staff	977 (1.26)
	Teacher	1023 (1.32)
	Active serviceman	74 (0.1)
	Policemen	135 (0.17)
	Private or individual business operator	7823 (10.13)
	Worker	2646 (3.42)
	Driver	1003 (1.3)
	Retired staff	7957 (10.3)
	Student	9802 (12.69)
	Child	8057 (10.43)
	Other	7514 (9.73)
**Marital status (n=77,271)^a^**		
	Unmarried	23,877 (30.9)
	Married	47,343 (61.27)
	Divorced	1662 (2.15)
	Widowed	3652 (4.73)
	Cohabitation	595 (0.77)
	Other	142 (0.18)
**Migrant worker (n=77,294)^a^**		
	Yes	8409 (10.88)
	No	68,885 (89.12)

^a^The missing values for age, gender, ethnicity, education, occupation, marital status, and migrant worker are 89, 12, 47, 43, 120, 110, and 87, respectively.

### Epidemiological Characteristics of Diseases

**Figure 1:**
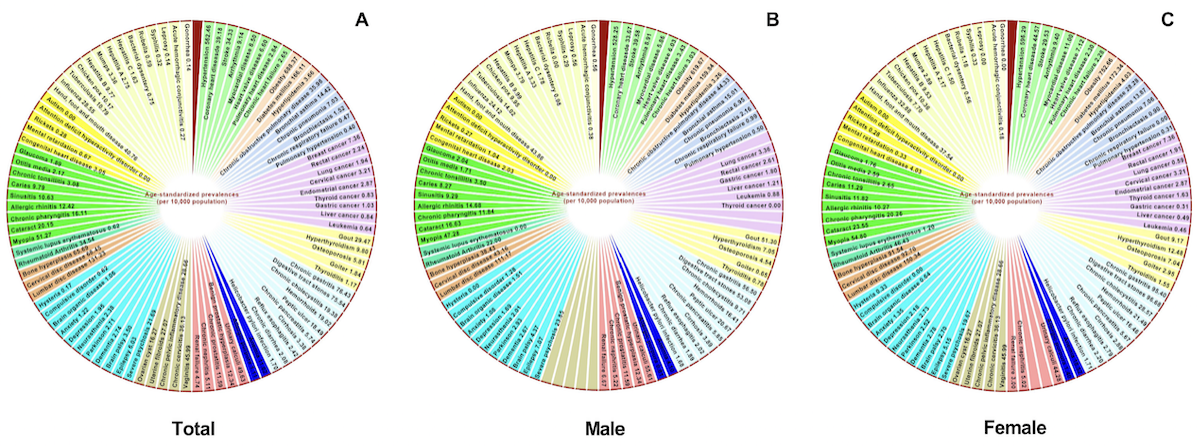
Epidemiological characteristics of diseases in Guiyang, China. All data are presented as age-standardized prevalence with 95% CIs (per 10,000 people). (A) Epidemiological characteristics of diseases. (B) Epidemiological characteristics of diseases in men. (C) Epidemiological characteristics of diseases in women. Higher-resolution version of this figure is available in Multimedia Appendix 7.

The epidemiological characteristics of diseases among residents in Guiyang, China, are shown in Figure 1 and Multimedia Appendices 5-6. The main diseases of residents are chronic NCDs, but there are still local epidemics of infectious diseases, such as hand, foot, and mouth disease. Based on our study findings in the natural population, the common NCDs in Guiyang residents are ranked from the most to least prevalent as follows: obesity, hypertension, diabetes mellitus, lumbar disc disease, chronic gastritis, digestive tract stones, cervical disc disease, bone hyperplasia, myopia, and urinary calculi*.* MTs in women are mostly breast cancer, cervical cancer, and endometrial cancer, whereas those in men are mainly lung cancer, rectal cancer, and gastric cancer.

### Gender Distribution Characteristics of NCDs and MTs

Table 2 shows the gender distribution characteristics of the top NCDs and MTs in Guiyang, China. Compared to women, the prevalences of obesity, hypertension, lumbar disc disease, chronic gastritis, digestive tract stones, cervical disc disease, and bone hyperplasia in men were significantly lower (all *P<*.001). However, the prevalence of urinary calculi in men was significantly higher than in women (*P=*.03). For MTs, the prevalence of thyroid cancer in women was higher than in men (*P=*.049), but prevalences of lung cancer and gastric cancer were lower in women than in men (*P=*.003 and *P*=.03, respectively). The disease rankings of NCDs and MTs in different genders are shown in Multimedia Appendices 8-9.

**Table 2:** Gender distribution characteristics of noncommunicable diseases and malignant tumors in residents of Guiyang, China. All data are presented as prevalence with 95% CIs (per 10,000 people).

Characteristic		Female, prevalence (95% CI)	Male, prevalence (95% CI)	*P* value
**Noncommunicable disease**				
	Obesity	752.66 (726.55-778.77)	619.57 (595.00-644.34)	<.001
	Hypertension	596.29 (573.04-619.53)	528.25 (505.61-550.90)	<.001
	Diabetes mellitus	172.34 (159.57-185.11)	159.84 (147.15-172.54)	.17
	Lumbar disc disease	150.34 (138.40-162.29)	111.17 (100.55-121.78)	<.001
	Chronic gastritis	95.40 (85.86-104.94)	56.50 (48.91-64.08)	<.001
	Digestive tract stones	96.68 (87.08-106.29)	53.08 (45.72-60.43)	<.001
	Cervical disc disease	92.70 (83.29-102.11)	43.16 (36.20-48.56)	<.001
	Bone hyperplasia	91.54 (82.19-100.89)	38.43 (30.70-46.13)	<.001
	Myopia	54.80 (47.56-62.05)	47.28 (40.34-54.22)	.13
	Urinary calculi	44.28 (39.31-54.04)	55.61 (48.08-63.14)	.03
**Malignant tumor**				
	Lung cancer	0.59 (0.00-1.35)	3.36 (1.51-5.22)	.003
	Gastric cancer	0.31 (0.00-0.86)	1.80 (0.44-3.16)	.03
	Liver cancer	0.49 (0.00-1.18)	1.21 (0.10-2.32)	.22
	Rectal cancer	1.91 (0.55-3.26)	2.61 (0.97-4.24)	.55
	Thyroid cancer	1.63 (0.37-2.88)	0.00 (0.00-0.00)	.049
	Leukemia	0.46 (0.00-1.12)	0.88 (0.00-1.82)	.61

### Age Distribution Characteristics of NCDs and MTs

The age distribution characteristics of diseases in Guiyang, China, are shown in Multimedia Appendices 8-10. The results clearly show that the spectrum of disease varies widely across age groups. Obesity (prevalence per 10,00 people: 385.69, 0-19 years; 462.58, 20-29 years; and 719.76, 30-39 years) and myopia (prevalence per 10,000 people: 60.78, 0-19 years; 84.22, 20-29 years; and 102.34, 30-39 years) are more common in people aged <40 years. The prevalences (per 10,000 people) of obesity (872.88, 40-49 years; 966.21, 50-59 years; 893.25, 60-69 years; and 869.43, ≥70 years); hypertension (467.74, 40-49 years; 1375.00, 50-59 years; 2556.89, 60-69 years; and 3730.31, ≥70 years); diabetes (150.62, 40-49 years; 477.00, 50-59 years; 718.87, 60-69 years; and 905.61, ≥70 years); and lumbar disc herniation (200.82, 40-49 years; 343.00, 50-59 years; 418.95, 60-69 years; and 420.16, ≥70 years) were higher in the age groups ≥40 years, and the prevalences increased with age except obesity. In the older adult population, in addition to the above diseases, coronary heart disease (162.21, 60-69 years and 445.71, ≥70 years, per 10,000 people); lumbar disc herniation (418.95, 60-69 years and 445.71, ≥70 years, per 10,000 people); and bone hyperplasia (324.43, 60-69 years and 363.38, ≥70 years, per 10,000 people) have gradually become the main diseases that threaten health. Hand, foot, and mouth disease (161.53 per 10,000 people) mainly occurs in school-age children. The analysis of the age distribution characteristics of the top 10 MTs in the study results showed that the prevalence of MTs was significantly different with increasing age (all *P<*.001).

### Regional Distribution Characteristics of NCDs and MTs

**Figure 2:**
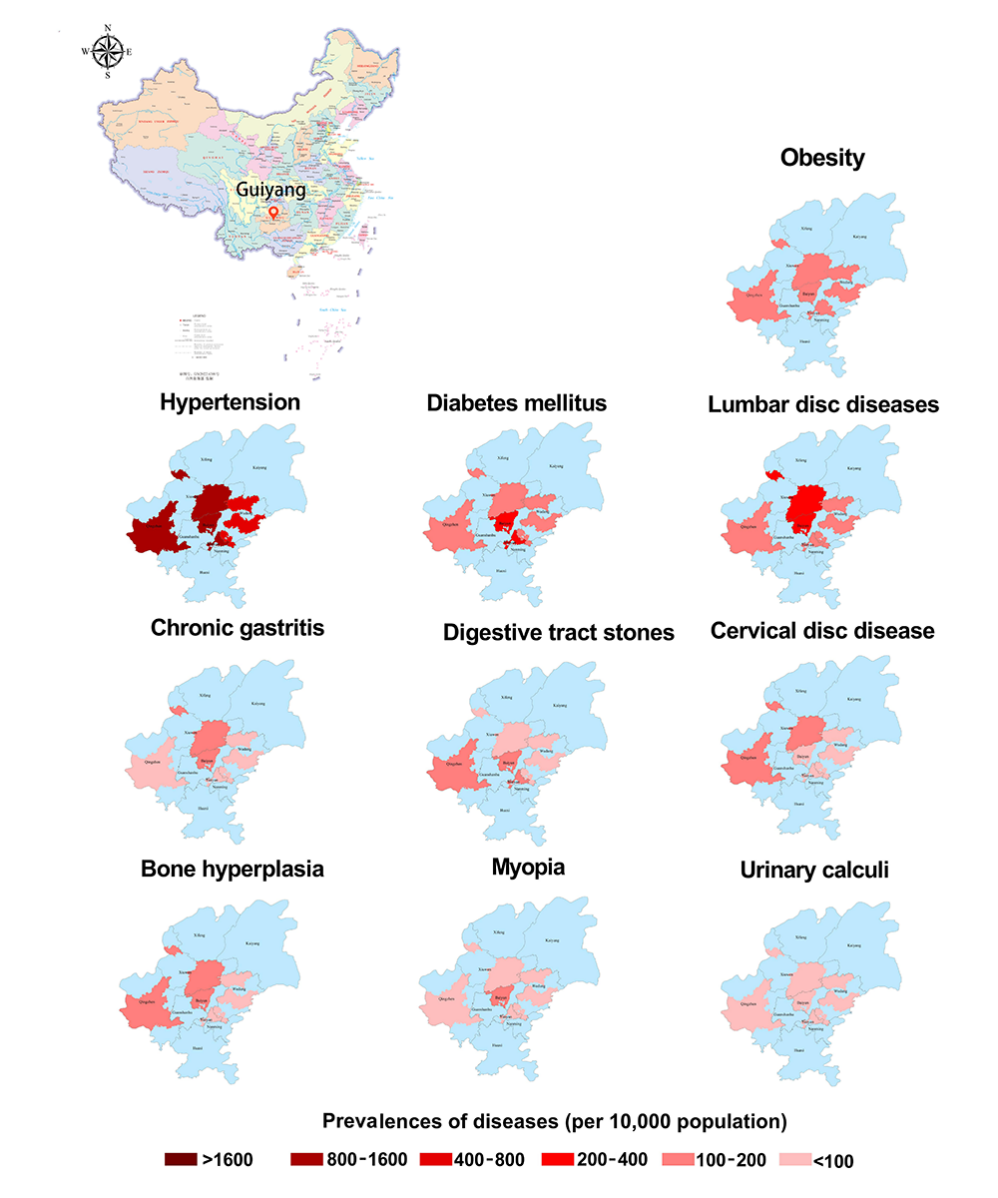
Regional distribution characteristics of noncommunicable diseases in Guiyang, China. Data illustrated in the choropleth maps are median prevalence estimates calculated from unadjusted logistic regressions. Higher-resolution version of this figure is available in Multimedia Appendix 7.

**Figure 3:**
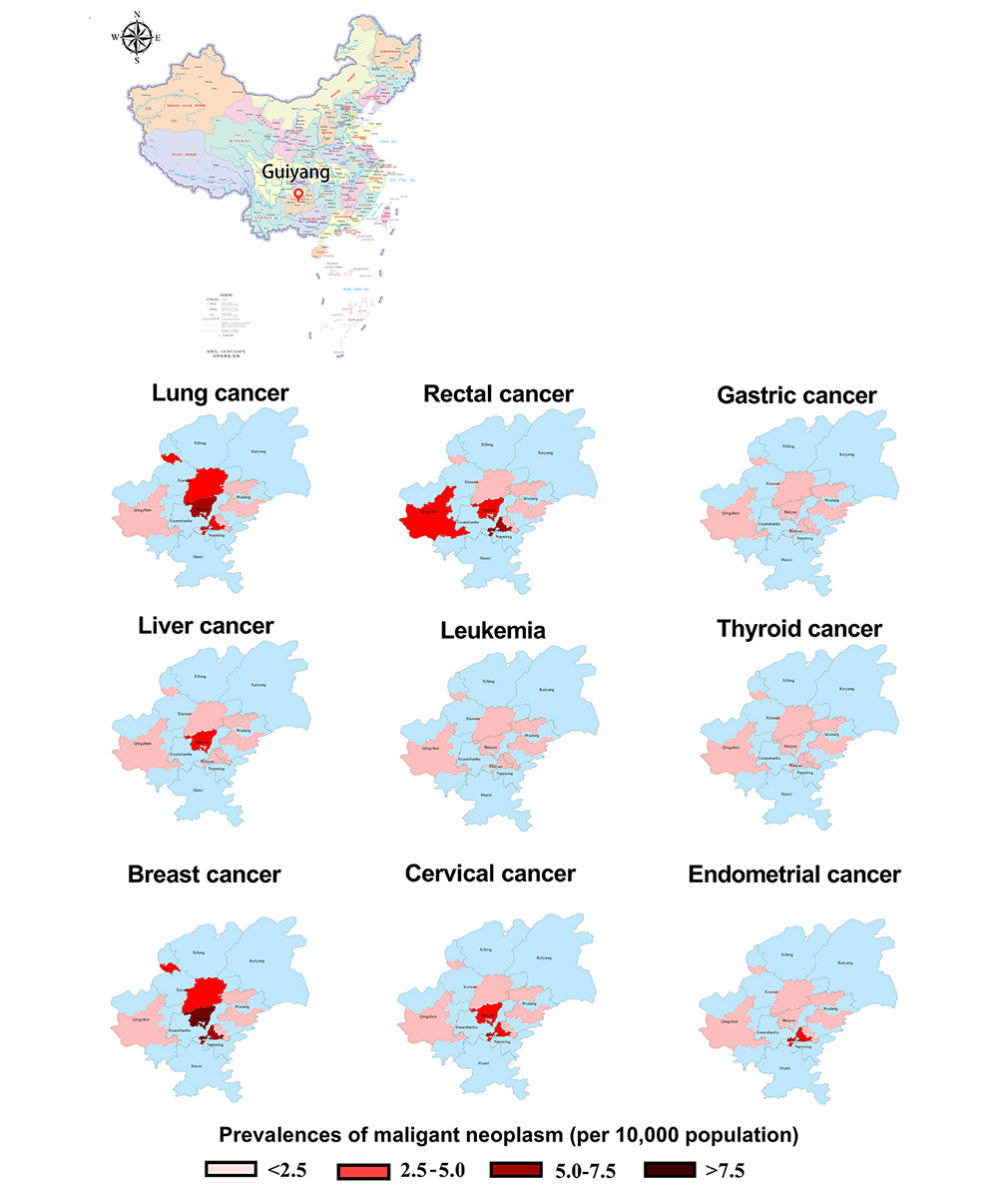
Regional distribution characteristics of malignant tumors in Guiyang, China. Data illustrated in the choropleth maps are median prevalence estimates calculated from unadjusted logistic regressions. Higher-resolution version of this figure is available in Multimedia Appendix 7.

Figure 2 shows the regional distribution characteristics of the common NCDs in Guiyang, China. From the statistics for single diseases, obesity is the disease with the highest prevalence in each district and county, followed by hypertension. Diabetes is the third most common of the NCDs in the Yunyan district of Guiyang, and lumbar disc herniation is most common in other districts. The high prevalence areas of hypertension and diabetes are mainly concentrated in the Baiyun and Yunyan districts and in Qingzhen county-city. Wudang and Huaxi districts and the counties of Xifeng and Kaiyang have lower prevalences of the abovementioned NCDs. As illustrated in Figure 3, Baiyun and Yunyan districts were the main high-prevalence areas of MTs, whereas the 4 regions of Xifeng (county), Kaiyang (county), Huaxi (district), and Qingzhen (county-city) had lower prevalences of MTs. The prevalences of lung cancer and breast cancer in Baiyun district are higher than in other districts or in counties, and the prevalences of rectal cancer, liver cancer, and cervical cancer are also higher in this area. The highest prevalence of MTs in Yunyan district is rectal cancer, and the prevalence of endometrial cancer is higher than in other districts or counties.

## Discussion

### Principal Findings

According to the latest statistics from the World Health Organization, in 2019, among the top 10 causes of death, 7 were NCDs; the total number of deaths from the 4 major NCDs was 33.2 million, including cardiovascular disease (17.9 million), cancer (9.3 million), diabetes (2 million), and chronic respiratory diseases (2.1 million) [14]. NCDs pose the greatest threat to human health in the modern world. From the perspective of the overall disease epidemiological characteristics, our study results show that the main diseases of residents in Guiyang are chronic NCDs, but there are still local epidemics of infectious diseases. These results are similar to those reported in other parts of the world [15-17]. From the perspective of population distribution characteristics, the epidemiological characteristics of diseases vary for different genders, ages, and regions. To effectively improve the health of the population, especially for people in different life stages, our findings suggest that disease prevention work should be targeted to different groups of people and that measures should be adapted to local conditions.

To better elucidate the epidemiological characteristics of NCDs among residents in Guiyang, the ranking of different diseases was analyzed based on standardized prevalences. The results demonstrated that obesity, hypertension, and diabetes mellitus are the top 3 NCDs in Guiyang. According to the latest estimates, 6.8% of children and adolescents (aged 5-19 years) worldwide were obese in 2016 [18]. Among adults aged ≥18 years, the age-standardized prevalence of obesity ranged from 4.7% in the Southeast Asia region to 28.6% in the region of the Americas [19]. Our results indicate that the prevalence of obesity is 6.88% for the entire population and 3.86% for the 0-20–year-old population. Globally, 59% of women and 49% of men with hypertension reported a previous diagnosis of hypertension in 2019 [20]. Our results are much lower than the world average, with a prevalence of hypertension of 5.96% in men and 5.28% in women. Although the global adult diabetes prevalence increased from 4.7% in 1980 to 8.5% in 2014 [21], the prevalence of diabetes in Guiyang remains low at 1.66%.

These findings are relatively encouraging—that the prevalences of NCDs and MTs in Guiyang are low. We speculate that the possible reasons are closely related to the good ecological environment and good air quality in Guiyang. A recent study of air pollution in Southwest China showed that the 3-year averages of particulate matter (PM)_1_, PM_2.5_, PM_10_, and NO_2_ in Guizhou were 26.3, 34.9, 56.2, and 22.0 μg·m^-3^, respectively, which were significantly lower than those in the municipality of Chongqing and Sichuan province [22]. Air pollution is significantly associated with NCDs [5]. However, overweight and obesity are major risk factors for NCDs such as cardiovascular disease, diabetes, and some cancers [23]. Moreover, hypertension and diabetes are the leading causes of death worldwide [6]. Therefore, strengthening health management and reasonably controlling weight, blood pressure, and blood glucose are still effective measures for preventing NCDs in Guiyang.

Our results demonstrate that MTs in women are mostly breast cancer, cervical cancer, and endometrial cancer, whereas in men, they are mainly lung cancer, rectal cancer, and gastric cancer. It is exciting to note that the prevalences of these tumors are significantly lower than the average age-standardized prevalence in the world (222 per 100,000) and China (225.4 per 100,000) [2]. Moreover, our MT findings appear to be at odds with the latest research reports around the world, as the latest cancer statistics [2] show that there is no gastric cancer among the top 10 most common MTs in men and no cervical cancer among the top 10 most common MTs in women. Previous studies [24,25] have shown that diet is closely related to the occurrence of gastric cancer, and a low-sodium diet can significantly reduce the incidence of gastric cancer. Several studies [26,27] have also found that human papillomavirus (HPV) infection is significantly associated with cervical cancer, and a small number of studies [28,29] have also revealed a high HPV infection rate among women in Guizhou. These studies suggest that the high rankings of gastric cancer and cervical cancer in Guiyang may be related to high-sodium diet and high HPV infection rate, respectively, in Guizhou. Therefore, implementing targeted preventive measures, such as disease screening and health education, for the high-risk groups for these key diseases may effectively control and prevent the occurrence of these diseases.

From a regional perspective on the prevalences of NCDs and MTs in Guiyang, the results clearly show that the prevalences of NCDs and MTs are relatively high in Yunyan district, Baiyun district, and other areas in central and western Guiyang. High population density, as well as rapid industrial and economic development are typical features of the Baiyun and Yunyan regions. These booming conditions may inevitably increase pollution sources and pollution loads, which may be the main reason for the high incidences of NCDs and MTs in these regions. Taking air pollution as an example, although air pollution affects people in all regions, all ages, and all social groups, it is likely to cause greater disease for those with high exposure and high susceptibility [30]. A previous study showed that from 1990 to 2019, the age-standardized mortality rate of cancer caused by PM_2.5_ had the highest increase [31], which may explain the high incidence of lung cancer in the Baiyun and Yunyan areas, because these areas are industrially developed areas in Guiyang, especially the nonferrous metal industry. Therefore, for the future in Guiyang, the management of populations in key areas should also be the focus of the prevention and control of NCDs and MTs. In addition, lifestyle choices, including smoking, drinking, sleeping, and eating habits, are related to the prevalence of many chronic diseases and MTs; regular physical exercise can reduce the occurrence of these diseases [32,33]. Therefore, actively advocating a healthy lifestyle, especially health education for high-risk groups, is an effective means for preventing disease.

### Strengths, Limitations, and Policy Recommendations

A strength of our study is that this is the first time the epidemiological characteristics of NCDs and MTs in Guiyang, China, were evaluated. Another strength is that we used a large-sample cross-sectional study. Our study also has limitations. Although we obtained the epidemiological characteristics of NCDs and MTs in Guiyang, including age, gender, and region, this study lacks comparison with previous baseline study data. In addition, we did not analyze possible factors that may underlie the high prevalences of NCDs and MTs, such as climate, air pollution, diet, lifestyle, and behavior. In addition, our study design required that the diagnosis of a specific disease was based on a clear medical history or a hospital diagnosis certificate. Thus, our study may have missed some patients, resulting in a slight underestimation of the prevalence and actual situation of a particular disease. Nevertheless, our study findings still have guiding significance for the epidemiology of diseases in Guiyang. Based on the current research results, the following policy recommendations are proposed: (1) combining high-risk groups with whole-population strategies to promote disease prevention; (2) strengthening multidepartmental collaboration and whole-society participation in health promotion, such as reducing unhealthy lifestyles and encouraging exercise; (3) making disease screening of key high-risk groups the starting point for improving the ability and efficiency of secondary prevention of diseases; (4) strengthening health education and actively guiding residents to use health services rationally; and (5) innovating the management of chronic diseases and improving the registration management of cancer patients.

### Conclusions

Overall, our study provides some evidence that several NCDs (obesity, hypertension, and diabetes) and MTs (women: breast cancer, cervical cancer, and endometrial cancer; men: lung cancer, rectal cancer, and gastric cancer) should be the focus for the prevention and control of chronic diseases in Guiyang in the future. In particular, the Baiyun and Yunyan districts of Guiyang, China, are the important regions to emphasize. The findings of this study may be of great value for preventing and controlling the occurrence and development of key chronic NCDs and MTs in Guiyang, as well as for improving the health of both urban and rural residents. Furthermore, our study results may aid decision makers in formulating more reasonable and effective resource allocation; preventive health policies; and solutions for NCDs, MTs, and related health inequalities.

## Appendix

Multimedia Appendix 1 Supplementary methods.

Multimedia Appendix 2 Distribution of the study participants.

Multimedia Appendix 3 The on-site investigation procedure.

Multimedia Appendix 4 Summary of measurements at the baseline survey.

Multimedia Appendix 5 The ranking of the top noncommunicable diseases (NCDs) in Guiyang, China.

Multimedia Appendix 6 The ranking of the top malignant tumors (MTs) in Guiyang, China.

Multimedia Appendix 7 Higher resolution versions of Figures 1-3.

Multimedia Appendix 8 Age distribution characteristics of diseases in Guiyang, China.

Multimedia Appendix 9 The prevalence of the top noncommunicable diseases (NCDs) in Guiyang, China, in different life cycles.

Multimedia Appendix 10 The prevalence of the top malignant tumors (MTs) in Guiyang, China, in different life cycles.

## Abbreviations

HPV: human papillomavirus
MT: malignant tumor
NCD: noncommunicable disease
PM: particulate matter

## Data Availability

The data sets generated during the current study are not publicly available but are available from the corresponding author upon reasonable request.
