# Supplementary material for: The Epidemiological Characteristics of Noncommunicable Diseases and Malignant Tumors in Guiyang, China: Cross-sectional Study
Source: JMIR Public Health Surveill. 2022 Oct 28;8(10):e36523. doi: 10.2196/36523 (PMC9652732; doi:10.2196/36523)
Supplement: Multimedia Appendix 1 [file publichealth_v8i10e36523_app1.pdf]

Supplementary methods

Study location

In this study, the investigation site is located in Guiyang City, Guizhou Province, China. The study location covered 10 districts or counties, including Yunyan District, Nanming District, Huaxi District, Guanshanhu District, Wudang District, Baiyun District, Qingzhen, Xifeng County, Xiuwen County and Kaiyang County. According to the sample size of the study design, there are 81,517 people in this survey. The survey population within the jurisdiction of each community health service center and township health center shall be allocated according to the population proportion of each region. The distribution of the study participants is presented in Table S1.

Sampling method

The method of multilevel stratified cluster sampling survey was adopted. Firstly, Guiyang was divided into three types of areas according to economic, geographical and other factors, the first type of areas (with good economic conditions, urban core areas, including Yunyan District and Nanming District), the second type of areas (with general economic conditions, non-urban core areas, including Baiyun District, Huaxi District, guanshanhu district and Wudang District) and the third type of areas (with relatively poor economic conditions, suburban District (counties), including Qingzhen, Xifeng County, Xiuwen County and Kaiyang County); then, according to the population ratio (1:2:2), using the method of simple random sampling, Yunyan District, Baiyun District, Wudang District, Qingzhen and Xiuwen County were selected as the first-class sampling clusters; finally, randomly selected townships or communities from the first-class sampling clusters, and conducted the household surveys in the communities, all family members in the selected households were the subjects of the survey.

Inclusion and exclusion criteria

Study selection criteria were: (1) informed consent was received from the participant; and (2) the participant belonged to the permanent population of Guiyang, China (defined as either a member of the migrant population who lived in the city for 6 of the past 12 months or a member of the local population who has been away from the city for less than 12 months). Individuals in different stages

of life were eligible, as there were no specific age requirements for study participation. Study exclusion criteria were: (1) inability to provide a unique national identity card; (2) presence of a severe physical or mental illness (such as schizophrenia or bipolar disorder); and (3) refusal to comply with research requirements. When an individual was ineligible, refused, or unavailable, a replacement household was selected from the initial list minus those households previously selected. These replacements ensured a sufficient sample size and representativeness of the data across the Guizhou.

Ethical considerations

The content, protocol, informed consent, and recruitment materials of this study were approved by the ethics committee of the affiliated hospital of Guizhou Medical University (NO.2017ER67), and all participants signed the informed consent.

Collaboration and field workflow

We conducted the baseline survey based on multi-agency collaboration consisting of academic institutions, centers for disease control (CDC), local clinical centers and local government. Each agency has a clear role in the process of investigation. Typically, the local government began the propaganda or publicity campaigns a few weeks before the formal investigation. Meanwhile, the residents were fully informed the benefits and requirements of participating into the study. Then the residents who were willing to participant can make an appointment with the local clinical center. On the scheduled day of investigation, participates were firstly asked to complete the enrolment process with their unique national identity cards, then checked eligibility and gave informed written consent.

Before the formal baseline recruitment, all the measurement instruments and the field workflow were pre-tested by a pilot study. The aim was to evaluate the clarity, feasibility, validity and reliability of questionnaire and other measurement instruments, and also the resources and personnel needed at each site. The formal baseline recruitment was then conducted. Besides the doctors and nurses from the local clinical centers, each site was normally allocated with 3 field coordinators, 15 interviewers and 3 field quality inspectors from academic institutions and CDCs. The whole clinic visits for each participant typically took 30–60 min to complete. The average daily

recruitment rate was 120–150 participants per site.

Questionnaire interview

A structured questionnaire was used to record information about demographic factors, health behavior, disease history of participants (see more details in Table S2). The information was collected by a face-to-face interview, implemented by well-trained interviewers recruited from the students with medical background in Guizhou Medical University. To avoid simple logic errors, our questionnaire had built-in strict logic limits. An information notification was also provided besides each question, which can give detailed instructions how to describe the questions properly. For a skilled interviewer, it usually took an average of 30 to 45 minutes to complete a questionnaire.

In addition to the questionnaire information, the duration time for each questionnaire was also collected. Those data were further used to assist the data quality control. At the same night after field investigation, we draw random samples of questionnaires to assess their data quality. The sampling scheme could ensure each interviewer be sampled at least once. The data quality assessment group consisted of 10 to 15 inspectors who were chosen from excellent interviewers. On the next day, the assessment report would be fed back to the interviewer to help them improve their interviewing skills. Questionnaires that were classified as unqualified would be excluded in the final analysis.

Clinical examination

We conducted the medical examinations mainly using the resources and personnel at local clinical centers. To unify the data standard across different sites, we implemented a standardized training for the doctors and nurses before the investigation. After training, there was a field simulation practice to test the training effect on the next morning, and a summing-up meeting in the following afternoon to optimize the workflow and fix encountered problems. We provided unified devices for each site and trained the local staffs to operate those devices. The CDC took the main responsibility of field quality control (QC) guided by a handbook and serval forms to monitor the key risk points. The main measures of QC included check whether the measurement instruments are in good condition, inspect whether the measurement in accordance with SOPs, random sample participants to re-test their results *etc*.

Physicians at the local clinical center conduct physical examinations (height, weight, blood pressure, waist circumference, hip circumference, etc.) of the subjects, and conduct clinical examinations and diagnoses. Except for self-declaration by the participants, all disease information must be recorded in the local health management system, or the participants can produce a hospital diagnosis certificate before they can be diagnosed.

The height measuring ruler and weight scale are calibrated with standard measuring tape and weight scale respectively before use. The data is normal within the range of ±2% and can be used for subsequent measurement. Otherwise, it needs to be calibrated before use. The height and fasting weight of the survey subjects were measured with a height measuring ruler and a weight scale. Body Mass Index (BMI) was derived from objectively measured height and weight. According to the health industry standard of the People's Republic of China "criteria of weight for adults"(WS/T 428-2013), all participants were stratified into one of four groups: underweight (BMI<18.5), normal weight (18.5≤BMI<24.0), overweight (24.0≤BMI<28.0) or obese (BMI≥28.0).

A soft ruler was used to measure the waist circumference and hip circumference of the survey subjects on an empty stomach, and each person was measured 3 times and the final average was obtained. When measuring waist circumference, the respondents stand naturally, shoulders are relaxed, and arms are crossed in front of their chests. The staff stands on the right side of the survey object. The specific positioning point of the tape measure should be placed at the level of the midpoint of the line connecting the anterior superior iliac spine and the lower border of the 12th rib. The data obtained by measuring the tape measure around the abdomen is the waist circumference. Choose the thickest part of your waist to measure. When measuring the hip circumference, the staff squatted on the right side of the subject's body, and after finding the maximum extension of the hip, wrap the soft ruler around the hip, and put it against the skin (avoid pressing) for reading. The final measurement result should be corrected according to the thickness of the clothing. For example, if men wear single-layer ordinary trousers, 2.5cm should be subtracted, and if women wear thin skirts, 1.0cm should be subtracted. According to the formula, the waist/hip ratio of the participants was calculated.

The electronic sphygmomanometer was calibrated before daily blood pressure measurement. Prepare a mercury sphygmomanometer, first measure the subject's blood pressure with the mercury sphygmomanometer 3 times and take the average value; then use the electronic sphygmomanometer

to measure the blood pressure of the same subject 3 times and take the average value, compare the data, if the electronic blood pressure value is within the mercury sphygmomanometer reading ± Within 5mmHg, the electronic sphygmomanometer is accurate and can be used for subsequent blood pressure measurement, otherwise it needs to be calibrated before use. Before the first measurement, the subjects were asked to sit still for 5 minutes. During the measurement, the subject sits opposite the investigator on the left side, with the left elbow flat on the table, and the feet flat and not crossed. Wrap the cuff around the left arm. The bottom of the cuff should be located 1cm-2cm above the inner side of the elbow joint of the upper arm, and the center of the cuff should be at the same level as the heart. Blood pressure and heart rate were measured by electronic sphygmomanometer. After each measurement, loosen the armband, allow the subject to move his arm a little, sit still for 1 minute, and take the next measurement. A total of 3 measurements were taken, with an interval of 1 minute between each measurement.

On-site investigation procedure

The on-site investigation procedure is shown in Figure S1.

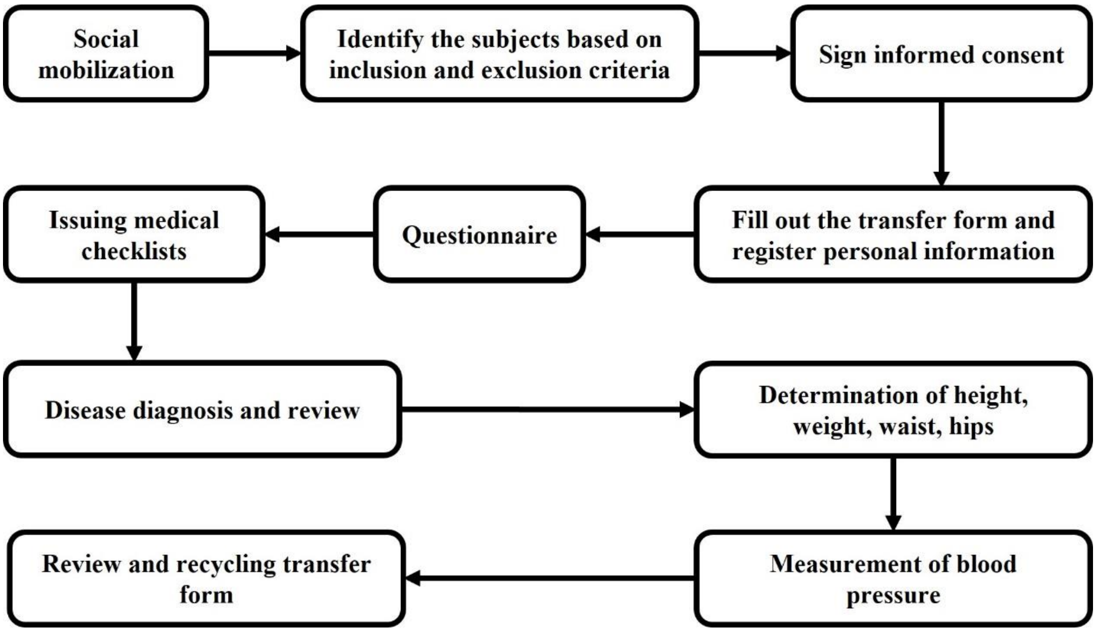

Figure S1 The on-site investigation procedure.
