# Supplementary material for: The Epidemiological Characteristics of Noncommunicable Diseases and Malignant Tumors in Guiyang, China: Cross-sectional Study
Source: JMIR Public Health Surveill. 2022 Oct 28;8(10):e36523. doi: 10.2196/36523 (PMC9652732; doi:10.2196/36523)
Supplement: Multimedia Appendix 2 [file publichealth_v8i10e36523_app2.pdf]

Table S1 Distribution of the study participants (n=81517)

Study location	Community or township	Number of people
**Yunyan district**	Xixia	3114
	Jinshi	2000
	Guiwu	2000
	Weiqing	2000
	Qianling	2000
	Zhonghua	2000
	Putuo	2000
	Zhaiji	2000
	Caiguan	2000
	Hetang	2000
	Dongshan	2000
	Zhonghuan	2000
	Qiandong	2000
	Jinya	2000
	Zhongtian	2000
	Shengfu	2000
**Baiyun district**	Dashandong	3000
	Yanshanhong	2000
	Hongyun	2000
	Maijia	2000

Study location	Community or township	Number of people
	Shawen	2000
**Wudang district**	Xintian	2000
	Xiaba	1893
	Chuangxin	2000
	Xinchang	2000
	Shuitian	2000
	Gaoxin	2000
	Yangchang	2000
**Qingzhen city**	Baihua	1310
	Hongxin	2000
	Hongta	2000
	Liuchang	2000
	Zhanjie	2000
	Liwei	2000
	Weicheng	2000
**Xiuwen county**	Longgang	2000
	Longchang	2000
	Zhazuo	2000
	Dashi Buyi	2000
	Jiuchang	2200

The total population in Yunyan district, Baiyun district, Wudang district, Qingzheng city, Xiuwen County are 960, 260, 380, 470, 250 thousand, respectively.
