# Supplementary figures and images for: The Epidemiological Characteristics of Noncommunicable Diseases and Malignant Tumors in Guiyang, China: Cross-sectional Study
Source: JMIR Public Health Surveill. 2022 Oct 28;8(10):e36523. doi: 10.2196/36523 (PMC9652732; doi:10.2196/36523)

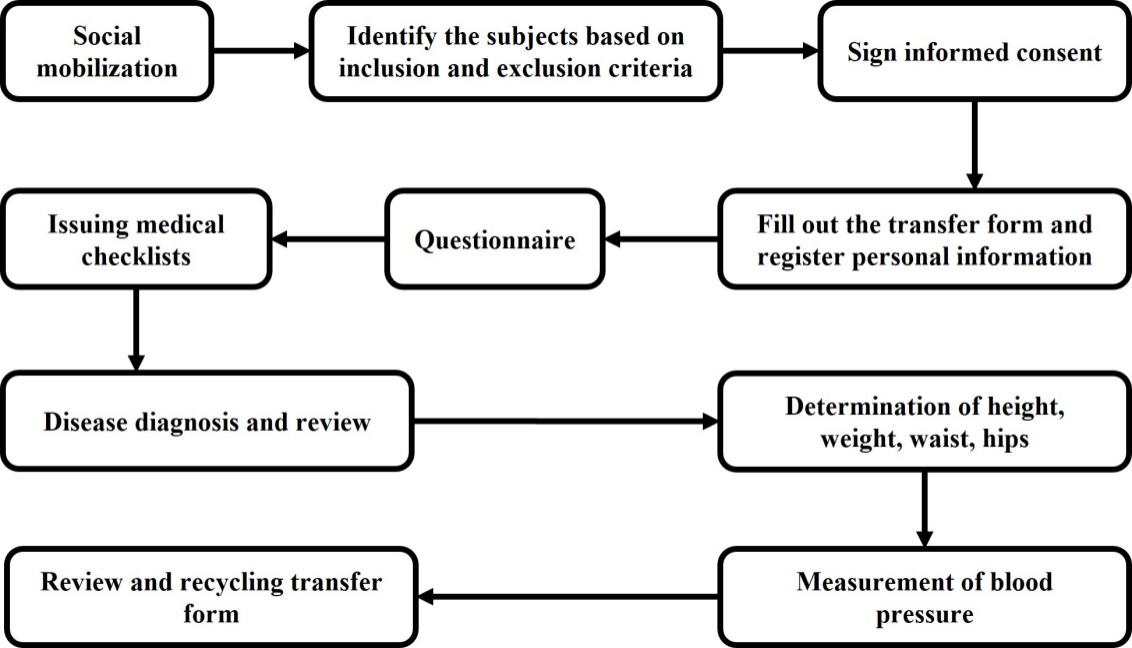

Supplement: Multimedia Appendix 3 [file publichealth_v8i10e36523_app3.pdf]

# Ranking of diseases for ages

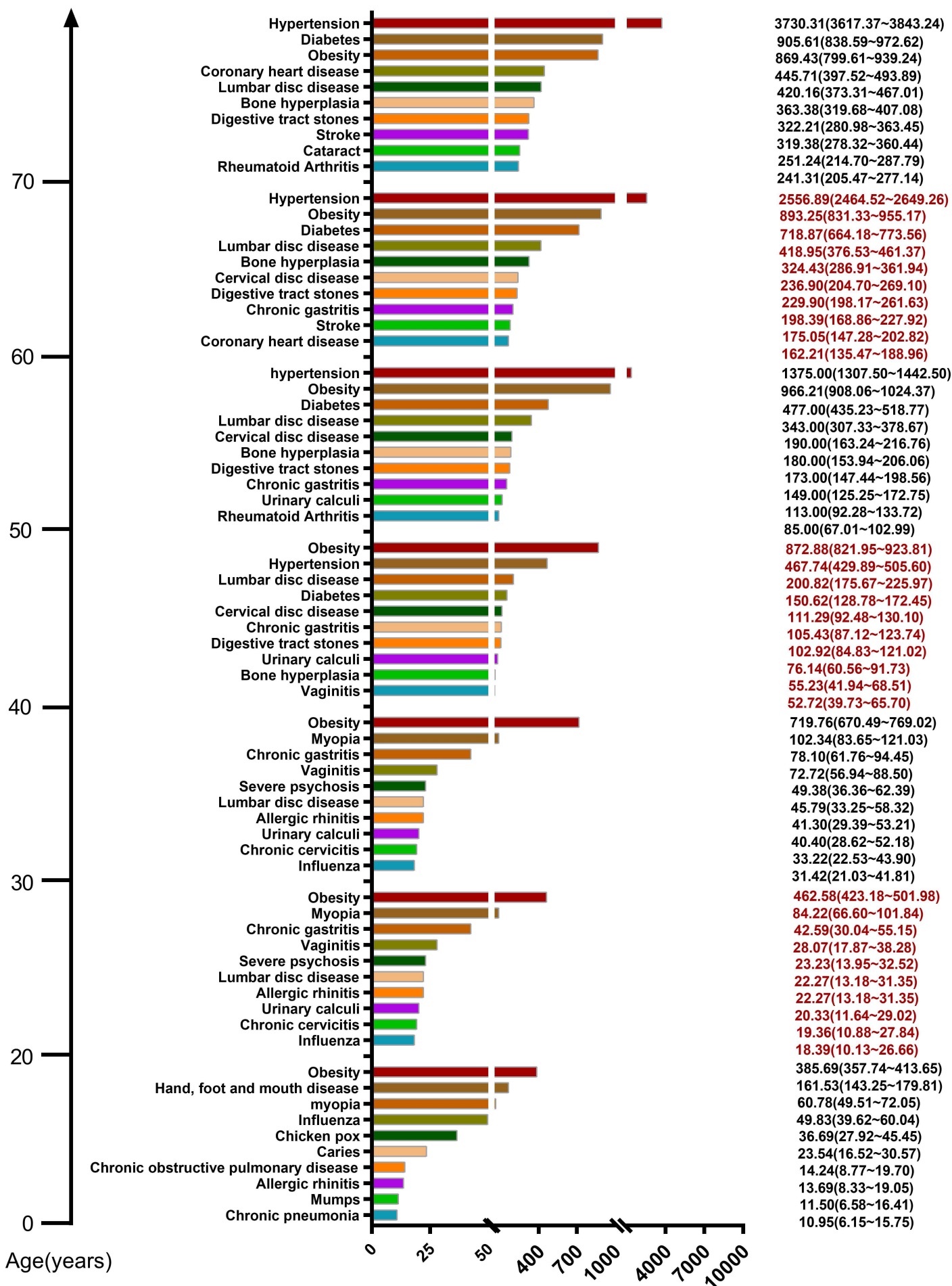

Supplement: Multimedia Appendix 8 [file publichealth_v8i10e36523_app8.pdf]
