# Supplementary material for: The Epidemiological Characteristics of Noncommunicable Diseases and Malignant Tumors in Guiyang, China: Cross-sectional Study
Source: JMIR Public Health Surveill. 2022 Oct 28;8(10):e36523. doi: 10.2196/36523 (PMC9652732; doi:10.2196/36523)
Supplement: Multimedia Appendix 4 [file publichealth_v8i10e36523_app4.pdf]

Table S2. Summary of measurements at the baseline survey

Measurements	No. of variables	Variables
**Structured questionnaire**		
Demographics	9	Name, National ID number, date of birth, sex, marital status, ethnic, education, occupation, contact information *etc.*
Health status	516	Disease name, whether or not you are sick, first visit institution, first visit time, treatment status, community follow-up management, *etc*.
Healthy behavior	29	Smoking, alcohol consumption, sleep, exercise and physical activity, diet *etc*.
**Clinical examination**		
Physical examination	4	Weight, height, waist circumference, hip circumference *etc*.
Blood pressure	7	whether to take antihypertensive drugs, diastolic blood pressure, systolic blood pressure *etc*.

The diseases including the respiratory system diseases (9 types), circulatory system diseases (6 types), endocrine system diseases (6 types), digestive system diseases (10 types), blood system diseases (8 types), female reproductive system diseases (6 types), neurological diseases Mental system diseases (12 types), motor system diseases (3 types), pediatric diseases (6 types), eye, ear, nose, and throat diseases (9 types), connective tissue diseases (2 types), malignant tumors (9 types).
