# Supplementary material for: The Epidemiological Characteristics of Noncommunicable Diseases and Malignant Tumors in Guiyang, China: Cross-sectional Study
Source: JMIR Public Health Surveill. 2022 Oct 28;8(10):e36523. doi: 10.2196/36523 (PMC9652732; doi:10.2196/36523)
Supplement: Multimedia Appendix 5 [file publichealth_v8i10e36523_app5.pdf]

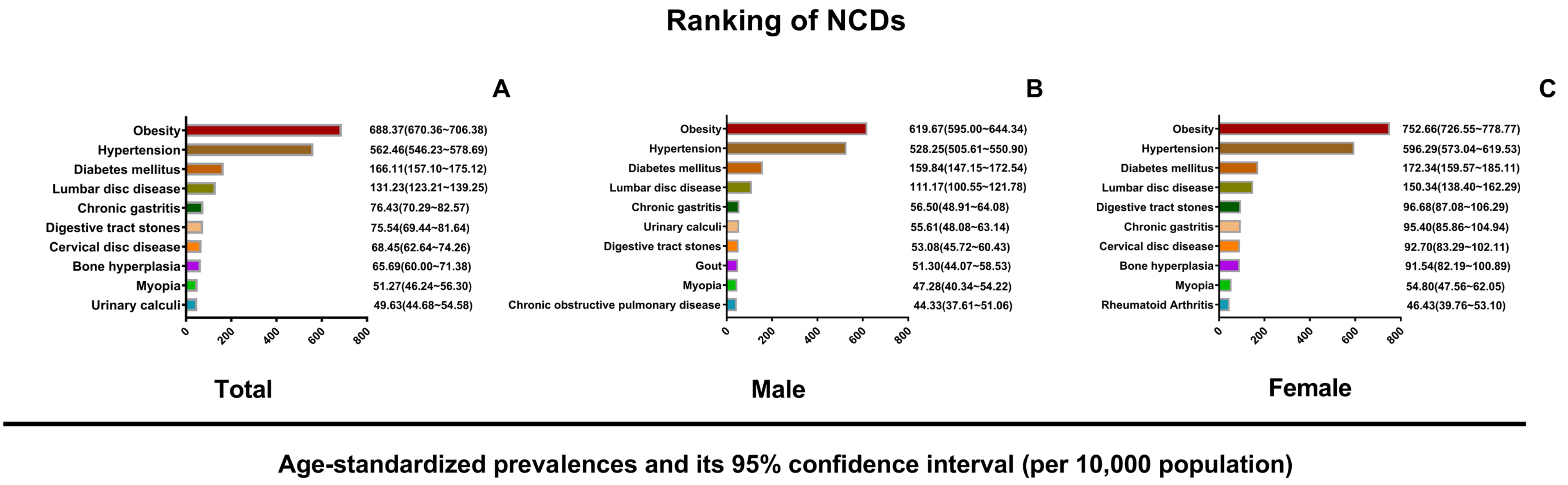
Ranking of NCDs
A
Obesity 688.37(670.36~706.38)
Hypertension 562.46(546.23~578.69)
Diabetes mellitus 166.11(157.10~175.12)
Lumbar disc disease 131.23(123.21~139.25)
Chronic gastritis 76.43(70.29~82.57)
Digestive tract stones 75.54(69.44~81.64)
Cervical disc disease 68.45(62.64~74.26)
Bone hyperplasia 65.69(60.00~71.38)
Myopia 51.27(46.24~56.30)
Urinary calculi 49.63(44.68~54.58)
0 200 400 600 800
Total
B
Obesity 619.67(595.00~644.34)
Hypertension 528.25(505.61~550.90)
Diabetes mellitus 159.84(147.15~172.54)
Lumbar disc disease 111.17(100.55~121.78)
Chronic gastritis 56.50(48.91~64.08)
Urinary calculi 55.61(48.08~63.14)
Digestive tract stones 53.08(45.72~60.43)
Gout 51.30(44.07~58.53)
Myopia 47.28(40.34~54.22)
Chronic obstructive pulmonary disease 44.33(37.61~51.06)
0 200 400 600 800
Male
C
Obesity 752.66(726.55~778.77)
Hypertension 596.29(573.04~619.53)
Diabetes mellitus 172.34(159.57~185.11)
Lumbar disc disease 150.34(138.40~162.29)
Digestive tract stones 96.68(87.08~106.29)
Chronic gastritis 95.40(85.86~104.94)
Cervical disc disease 92.70(83.29~102.11)
Bone hyperplasia 91.54(82.19~100.89)
Myopia 54.80(47.56~62.05)
Rheumatoid Arthritis 46.43(39.76~53.10)
0 200 400 600 800
Female
Age-standardized prevalences and its 95% confidence interval (per 10,000 population)
