# Supplementary material for: The Epidemiological Characteristics of Noncommunicable Diseases and Malignant Tumors in Guiyang, China: Cross-sectional Study
Source: JMIR Public Health Surveill. 2022 Oct 28;8(10):e36523. doi: 10.2196/36523 (PMC9652732; doi:10.2196/36523)
Supplement: Multimedia Appendix 6 [file publichealth_v8i10e36523_app6.pdf]

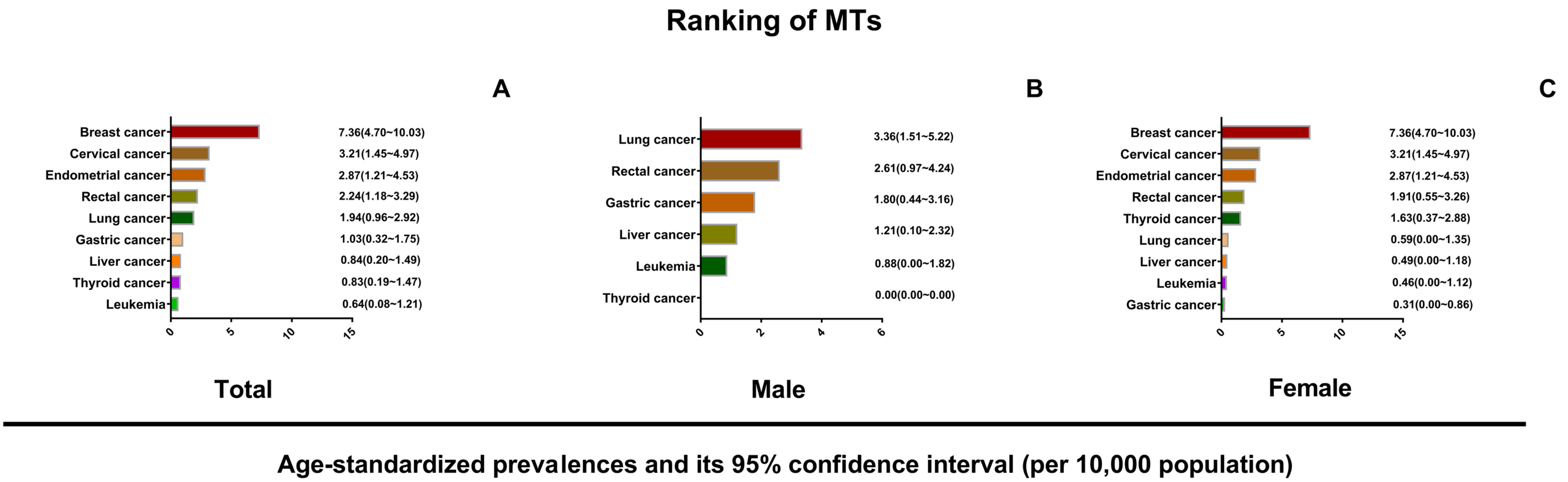
Ranking of MTs
A
B
C
Breast cancer
Cervical cancer
Endometrial cancer
Rectal cancer
Lung cancer
Gastric cancer
Liver cancer
Thyroid cancer
Leukemia
7.36(4.70~10.03)
3.21(1.45~4.97)
2.87(1.21~4.53)
2.24(1.18~3.29)
1.94(0.96~2.92)
1.03(0.32~1.75)
0.84(0.20~1.49)
0.83(0.19~1.47)
0.64(0.08~1.21)
0
5
10
15
Total
Lung cancer
Rectal cancer
Gastric cancer
Liver cancer
Leukemia
Thyroid cancer
3.36(1.51~5.22)
2.61(0.97~4.24)
1.80(0.44~3.16)
1.21(0.10~2.32)
0.88(0.00~1.82)
0.00(0.00~0.00)
0
2
4
6
Male
Breast cancer
Cervical cancer
Endometrial cancer
Rectal cancer
Thyroid cancer
Lung cancer
Liver cancer
Leukemia
Gastric cancer
7.36(4.70~10.03)
3.21(1.45~4.97)
2.87(1.21~4.53)
1.91(0.55~3.26)
1.63(0.37~2.88)
0.59(0.00~1.35)
0.49(0.00~1.18)
0.46(0.00~1.12)
0.31(0.00~0.86)
0
5
10
15
Female
Age-standardized prevalences and its 95% confidence interval (per 10,000 population)
