# Supplementary material for: The Epidemiological Characteristics of Noncommunicable Diseases and Malignant Tumors in Guiyang, China: Cross-sectional Study
Source: JMIR Public Health Surveill. 2022 Oct 28;8(10):e36523. doi: 10.2196/36523 (PMC9652732; doi:10.2196/36523)
Supplement: Multimedia Appendix 7 [file publichealth_v8i10e36523_fig.pdf]

Epidemiological characteristics of diseases among residents in Guiyang, China

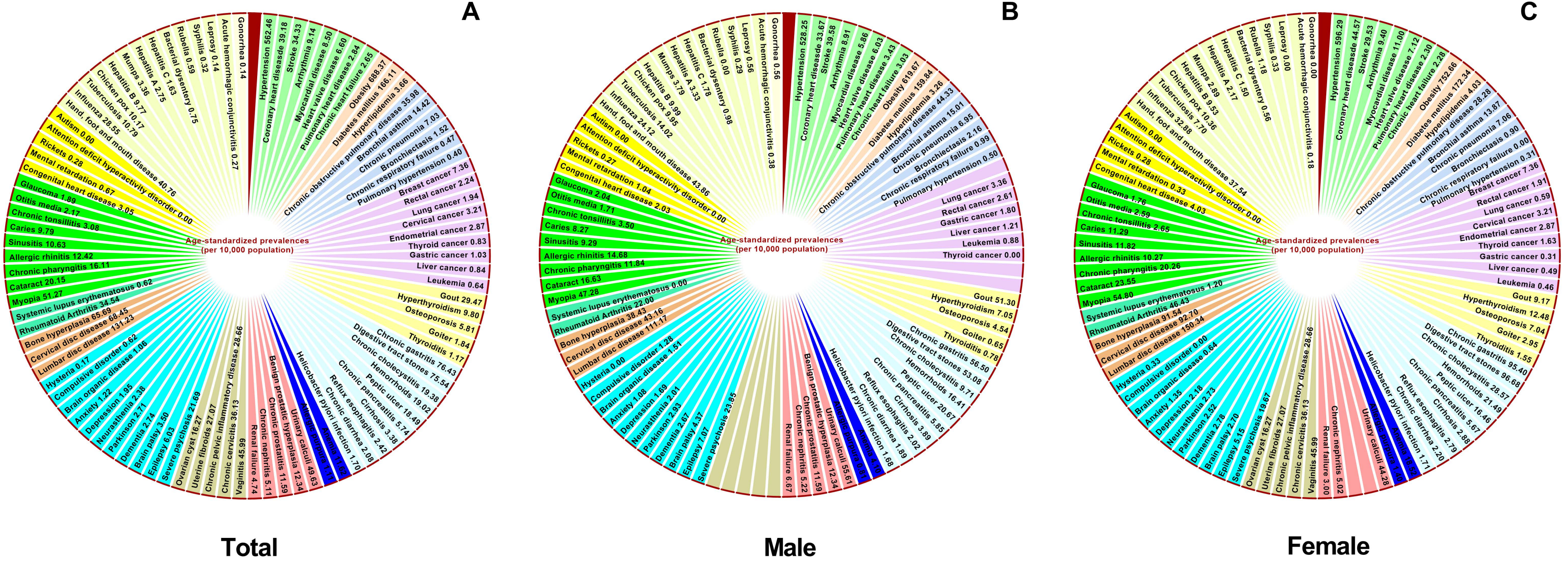

Age-standardized prevalences and its 95% confidence interval (per 10,000 population)

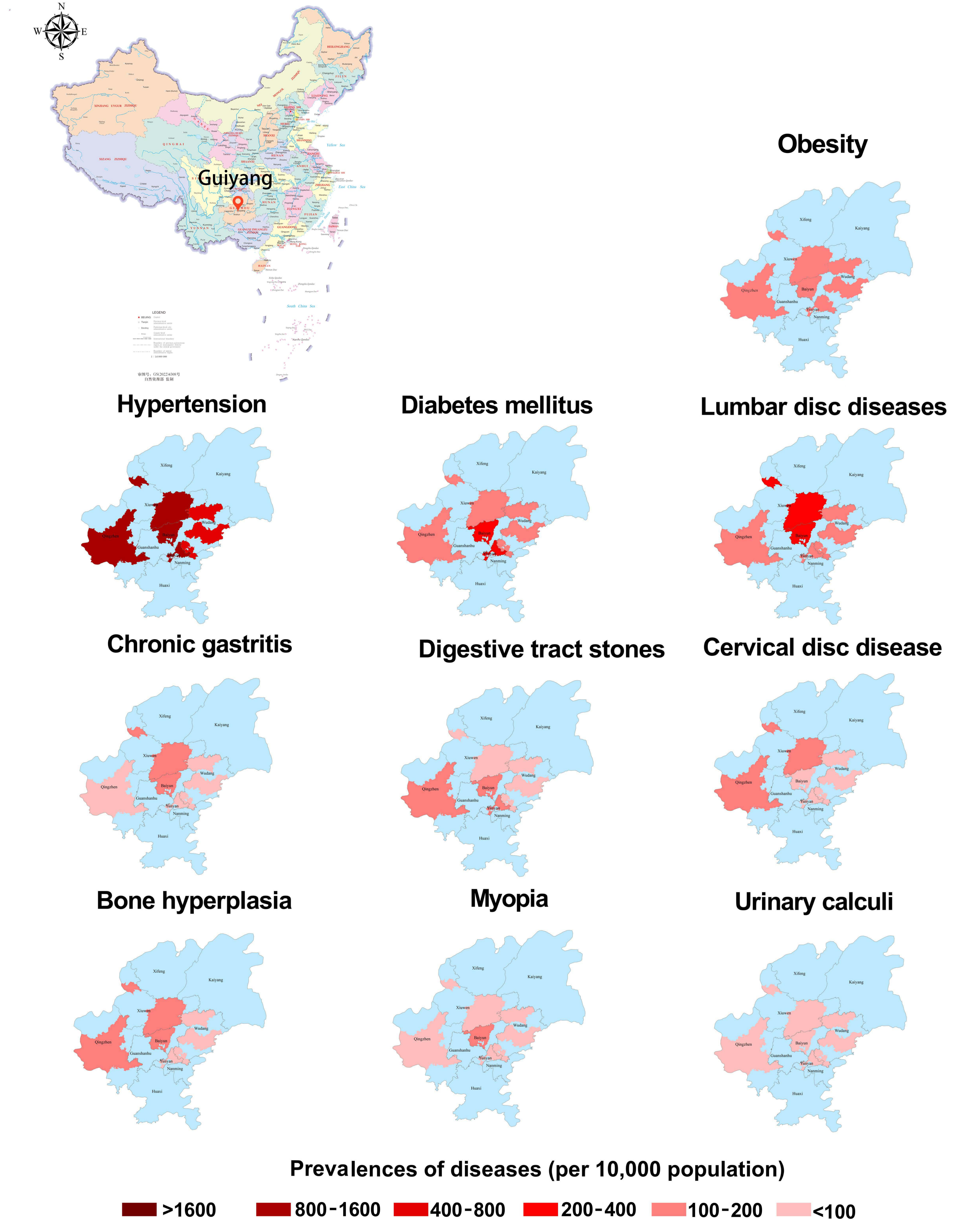

Guiyang
Obesity
Hypertension
Diabetes mellitus
Lumbar disc diseases
Chronic gastritis
Digestive tract stones
Cervical disc disease
Bone hyperplasia
Myopia
Urinary calculi
Xifeng
Kaiyang
Xiuwen
Qingzhen
Baiyun
Wudang
Guanshanhu
Yunyan
Nanming
Huaxi
Prevalences of diseases (per 10,000 population)
>1600
800-1600
400-800
200-400
100-200
<100

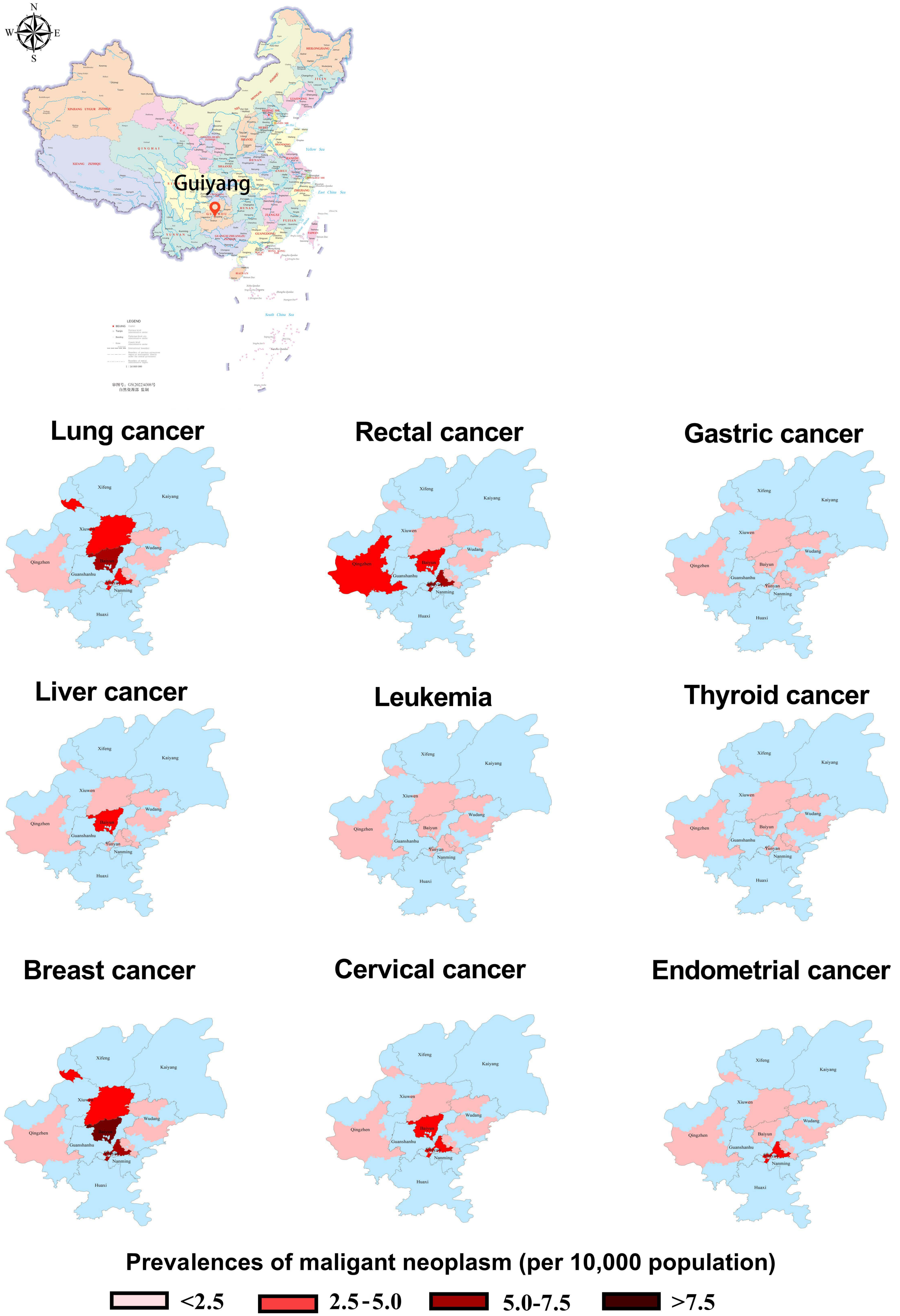
N
S
W
E
Guiyang
Lung cancer
Rectal cancer
Gastric cancer
Liver cancer
Leukemia
Thyroid cancer
Breast cancer
Cervical cancer
Endometrial cancer
Xifeng
Kaiyang
Xiuwen
Wudang
Qingzhen
Baiyun
Guanshanhu
Yunyan
Nanming
Huaxi
Prevalences of maligant neoplasm (per 10,000 population)
<2.5
2.5-5.0
5.0-7.5
>7.5
