# Supplementary material for: The Epidemiological Characteristics of Noncommunicable Diseases and Malignant Tumors in Guiyang, China: Cross-sectional Study
Source: JMIR Public Health Surveill. 2022 Oct 28;8(10):e36523. doi: 10.2196/36523 (PMC9652732; doi:10.2196/36523)
Supplement: Multimedia Appendix 9 [file publichealth_v8i10e36523_app9.pdf]

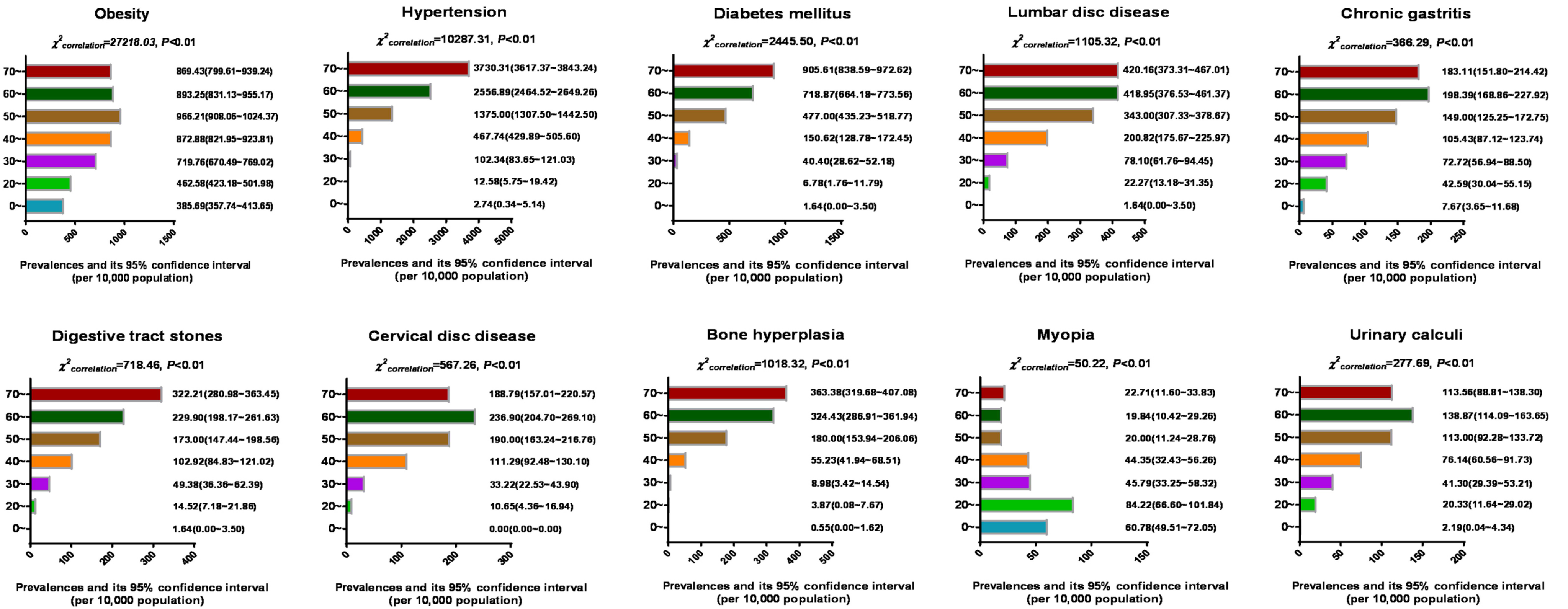
Obesity
χ²correlation=27218.03, P<0.01
70~ 869.43(799.61~939.24)
60~ 893.25(831.13~955.17)
50~ 966.21(908.06~1024.37)
40~ 872.88(821.95~923.81)
30~ 719.76(670.49~769.02)
20~ 462.58(423.18~501.98)
0~ 385.69(357.74~413.65)
Prevalences and its 95% confidence interval (per 10,000 population)
Hypertension
χ²correlation=10287.31, P<0.01
70~ 3730.31(3617.37~3843.24)
60~ 2556.89(2464.52~2649.26)
50~ 1375.00(1307.50~1442.50)
40~ 467.74(429.89~505.60)
30~ 102.34(83.65~121.03)
20~ 12.58(5.75~19.42)
0~ 2.74(0.34~5.14)
Prevalences and its 95% confidence interval (per 10,000 population)
Diabetes mellitus
χ²correlation=2445.50, P<0.01
70~ 905.61(838.59~972.62)
60~ 718.87(664.18~773.56)
50~ 477.00(435.23~518.77)
40~ 150.62(128.78~172.45)
30~ 40.40(28.62~52.18)
20~ 6.78(1.76~11.79)
0~ 1.64(0.00~3.50)
Prevalences and its 95% confidence interval (per 10,000 population)
Lumbar disc disease
χ²correlation=1105.32, P<0.01
70~ 420.16(373.31~467.01)
60~ 418.95(376.53~461.37)
50~ 343.00(307.33~378.67)
40~ 200.82(175.67~225.97)
30~ 78.10(61.76~94.45)
20~ 22.27(13.18~31.35)
0~ 1.64(0.00~3.50)
Prevalences and its 95% confidence interval (per 10,000 population)
Chronic gastritis
χ²correlation=366.29, P<0.01
70~ 183.11(151.80~214.42)
60~ 198.39(168.86~227.92)
50~ 149.00(125.25~172.75)
40~ 105.43(87.12~123.74)
30~ 72.72(56.94~88.50)
20~ 42.59(30.04~55.15)
0~ 7.67(3.65~11.68)
Prevalences and its 95% confidence interval (per 10,000 population)
Digestive tract stones
χ²correlation=718.46, P<0.01
70~ 322.21(280.98~363.45)
60~ 229.90(198.17~261.63)
50~ 173.00(147.44~198.56)
40~ 102.92(84.83~121.02)
30~ 49.38(36.36~62.39)
20~ 14.52(7.18~21.86)
0~ 1.64(0.00~3.50)
Prevalences and its 95% confidence interval (per 10,000 population)
Cervical disc disease
χ²correlation=567.26, P<0.01
70~ 188.79(157.01~220.57)
60~ 236.90(204.70~269.10)
50~ 190.00(163.24~216.76)
40~ 111.29(92.48~130.10)
30~ 33.22(22.53~43.90)
20~ 10.65(4.36~16.94)
0~ 0.00(0.00~0.00)
Prevalences and its 95% confidence interval (per 10,000 population)
Bone hyperplasia
χ²correlation=1018.32, P<0.01
70~ 363.38(319.68~407.08)
60~ 324.43(286.91~361.94)
50~ 180.00(153.94~206.06)
40~ 55.23(41.94~68.51)
30~ 8.98(3.42~14.54)
20~ 3.87(0.08~7.67)
0~ 0.55(0.00~1.62)
Prevalences and its 95% confidence interval (per 10,000 population)
Myopia
χ²correlation=50.22, P<0.01
70~ 22.71(11.60~33.83)
60~ 19.84(10.42~29.26)
50~ 20.00(11.24~28.76)
40~ 44.35(32.43~56.26)
30~ 45.79(33.25~58.32)
20~ 84.22(66.60~101.84)
0~ 60.78(49.51~72.05)
Prevalences and its 95% confidence interval (per 10,000 population)
Urinary calculi
χ²correlation=277.69, P<0.01
70~ 113.56(88.81~138.30)
60~ 138.87(114.09~163.65)
50~ 113.00(92.28~133.72)
40~ 76.14(60.56~91.73)
30~ 41.30(29.39~53.21)
20~ 20.33(11.64~29.02)
0~ 2.19(0.04~4.34)
Prevalences and its 95% confidence interval (per 10,000 population)
