# Supplementary material for: The Epidemiological Characteristics of Noncommunicable Diseases and Malignant Tumors in Guiyang, China: Cross-sectional Study
Source: JMIR Public Health Surveill. 2022 Oct 28;8(10):e36523. doi: 10.2196/36523 (PMC9652732; doi:10.2196/36523)
Supplement: Multimedia Appendix 10 [file publichealth_v8i10e36523_app10.pdf]

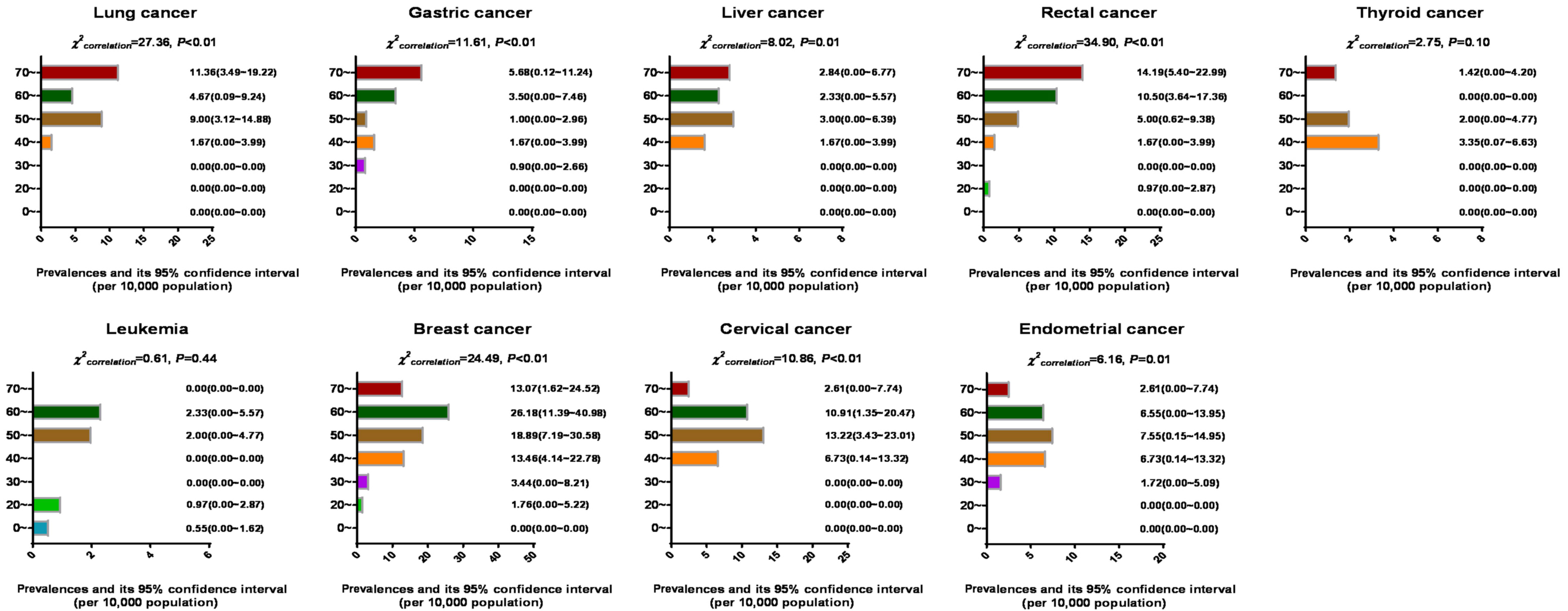

Lung cancer
χ2correlation=27.36, P<0.01
11.36(3.49~19.22)
4.67(0.09~9.24)
9.00(3.12~14.88)
1.67(0.00~3.99)
0.00(0.00~0.00)
0.00(0.00~0.00)
0.00(0.00~0.00)
Gastric cancer
χ2correlation=11.61, P<0.01
5.68(0.12~11.24)
3.50(0.00~7.46)
1.00(0.00~2.96)
1.67(0.00~3.99)
0.90(0.00~2.66)
0.00(0.00~0.00)
0.00(0.00~0.00)
Liver cancer
χ2correlation=8.02, P=0.01
2.84(0.00~6.77)
2.33(0.00~5.57)
3.00(0.00~6.39)
1.67(0.00~3.99)
0.00(0.00~0.00)
0.00(0.00~0.00)
0.00(0.00~0.00)
Rectal cancer
χ2correlation=34.90, P<0.01
14.19(5.40~22.99)
10.50(3.64~17.36)
5.00(0.62~9.38)
1.67(0.00~3.99)
0.00(0.00~0.00)
0.97(0.00~2.87)
0.00(0.00~0.00)
Thyroid cancer
χ2correlation=2.75, P=0.10
1.42(0.00~4.20)
0.00(0.00~0.00)
2.00(0.00~4.77)
3.35(0.07~6.63)
0.00(0.00~0.00)
0.00(0.00~0.00)
0.00(0.00~0.00)
Leukemia
χ2correlation=0.61, P=0.44
0.00(0.00~0.00)
2.33(0.00~5.57)
2.00(0.00~4.77)
0.00(0.00~0.00)
0.00(0.00~0.00)
0.97(0.00~2.87)
0.55(0.00~1.62)
Breast cancer
χ2correlation=24.49, P<0.01
13.07(1.62~24.52)
26.18(11.39~40.98)
18.89(7.19~30.58)
13.46(4.14~22.78)
3.44(0.00~8.21)
1.76(0.00~5.22)
0.00(0.00~0.00)
Cervical cancer
χ2correlation=10.86, P<0.01
2.61(0.00~7.74)
10.91(1.35~20.47)
13.22(3.43~23.01)
6.73(0.14~13.32)
0.00(0.00~0.00)
0.00(0.00~0.00)
0.00(0.00~0.00)
Endometrial cancer
χ2correlation=6.16, P=0.01
2.61(0.00~7.74)
6.55(0.00~13.95)
7.55(0.15~14.95)
6.73(0.14~13.32)
1.72(0.00~5.09)
0.00(0.00~0.00)
0.00(0.00~0.00)
70~
60~
50~
40~
30~
20~
0~
Prevalences and its 95% confidence interval
(per 10,000 population)
